# Digital Twin-Assisted Beamforming for Millimeter Wave Massive MIMO

**DOI:** 10.3390/s26185715

**Published:** 2026-09-09

**Authors:** Ke Xu, Weiqiang Wu

**Affiliations:** School of Integrated Circuits, Shenzhen Polytechnic University, Shenzhen 518055, China; xuke1991@szpu.edu.cn

**Keywords:** digital twin, massive MIMO, beamforming

## Abstract

Millimeter wave (mmWave) Massive MIMO is a cornerstone technology for sixth-generation (6G) wireless networks, providing the directional gain necessary to overcome high path loss. However, the acquisition of high-fidelity Channel State Information (CSI) and the associated beamforming overhead remain significant bottlenecks, particularly in dynamic environments with frequent blockages. In this paper, we propose a fast and robust beamforming strategy enabled by a digital twin (DT) framework. Specifically, we develop a Conditional Generative Adversarial Network (cGAN)-based DT module that serves as a high-fidelity virtual surrogate for site-specific ray-tracing. By processing environmental 3D geometry and dynamic obstacle data, the cGAN predicts real-time Beam-Power Maps (BPM) with minimal computational latency. Building upon these predictions, we introduce a Graph Neural Network (GNN)-based resource allocation agent that models the network as a spatial interference graph to perform coordination and power control. Numerical results demonstrate that our proposed DT-assisted approach significantly reduces online interaction overhead by shifting the computational burden of ray-tracing to an offline generative phase. Furthermore, the framework achieves superior sum-rate performance and link robustness under dynamic blockages compared to conventional deep learning and heuristic benchmarks.

## 1. Introduction

Future wireless communication systems rely on millimeter wave (mmWave) Massive MIMO as a critical enabler for achieving unprecedented spectral efficiency [1]. By utilizing large-scale antenna arrays at the base station (BS), Massive MIMO systems can generate highly directional beams to compensate for the severe path loss and atmospheric attenuation characteristic of the mmWave spectrum [2]. Despite these advantages, the performance of mmWave links is highly susceptible to environmental dynamics; even minor movements of mobile obstacles, such as vehicles or pedestrians, can cause abrupt line-of-sight (LoS) blockages and catastrophic signal degradation [3].

To maintain robust connectivity, traditional systems rely on periodic beam sweeping or frequent pilot-based CSI feedback. However, in Massive MIMO configurations, the large dimensionality of the antenna arrays results in a prohibitive training overhead that often exceeds the channel coherence time. Consequently, recent research has pivoted toward deep learning (DL)-based beamforming to automate the mapping between environmental features and optimal beam vectors [4]. Various architectures, including deep neural networks (DNNs) and deep reinforcement learning (DRL), have shown promise in learning complex spatial patterns and tracking user mobility without explicit CSI [5,6,7]. Nevertheless, these “black-box” DL models often suffer from a significant “reality gap”, requiring massive amounts of real-world interaction data to converge, which is both costly and operationally risky in live network deployments.

Digital twin (DT) technology has emerged as a transformative solution to bridge this gap by creating high-fidelity virtual replicas of the physical radio environment [8]. By integrating 3D geometric models, material properties, and real-time sensor data, a DT can simulate electromagnetic wave propagation using high-precision ray-tracing (RT) engines [9]. Recent industry guidelines, such as the Beyond 5G White Paper on AI and digital twins [10], highlight the potential of replacing these computationally heavy RT engines with AI/ML models to predict radio propagation. While recent literature has increasingly explored DT-assisted architectures to bridge the reality gap in mmWave communications, existing works predominantly focus on isolated, single-user beam prediction and still rely heavily on online wave-calculations or reactive beam measurement reports. Consequently, they suffer from prohibitive computational latency that often exceeds the channel coherence time of dynamic links. The specific methodological novelty of our proposed framework over state-of-the-art DT solutions and conventional guidelines such as [10] is two-fold. First, we completely shift the heavy computational burden of physical ray-tracing to an offline generative phase using a cGAN, enabling near-instantaneous, “learned” ray-tracing during online inference. Second, unlike conventional DT-assisted DL models that stop at beam prediction, our architecture feeds this high-fidelity spatial intelligence into a downstream GNN-based resource allocation agent. By modeling the multi-user network as a spatial interference graph, the GNN performs proactive over-the-air coordination and power control—an interference-aware capability lacking in current DT-assisted beamforming literature.

In this work, we propose a novel DT-assisted framework that leverages generative AI and graph representation learning for real-time mmWave resource allocation. Our primary contributions are as follows:

- Unlike conventional frameworks that calculate propagation online, our system shifts the computational burden to an offline generative phase using a Conditional Generative Adversarial Network (cGAN). This allows the DT to instantaneously hallucinate high-fidelity Beam-Power Maps (BPM) during online inference.
- We extend beyond isolated single-user prediction by feeding the cGAN’s spatial intelligence into a downstream Graph Neural Network (GNN) agent. By modeling the multi-user environment as a spatial interference graph, the GNN performs proactive over-the-air coordination and multi-user power control.
- Rather than reacting to beam failure reports, our framework embeds real-time dynamic obstacle coordinates into the DT’s multi-layered condition tensor. This geometric intelligence enables the system to seamlessly switch to non-line-of-sight (NLOS) reflection paths before a physical blockage severs the primary link.

Numerical evaluations demonstrate that the proposed framework achieves a remarkable reduction in online interaction overhead while maintaining a sum-rate performance that closely approaches the theoretical optimum of exhaustive ray-tracing search.

The remainder of this paper is organized as follows. Section 2 presents the system and channel model, detailing the bifurcation between the physical entity and the digital twin environment. Section 3 formulates the sum-rate maximization problem and identifies the computational latency constraints inherent in traditional ray-tracing. Section 4 introduces the proposed cGAN-assisted methodology, elaborating on the generative architecture for the digital twin surrogate and the offline training phase. Section 5 describes the intelligent resource allocation strategies, including context-aware beam pruning and GNN-based interference management. Section 6 provides a comprehensive numerical analysis and performance discussion. Finally, Section 7 concludes the paper.

## 2. System and Channel Model

**Figure 1:**
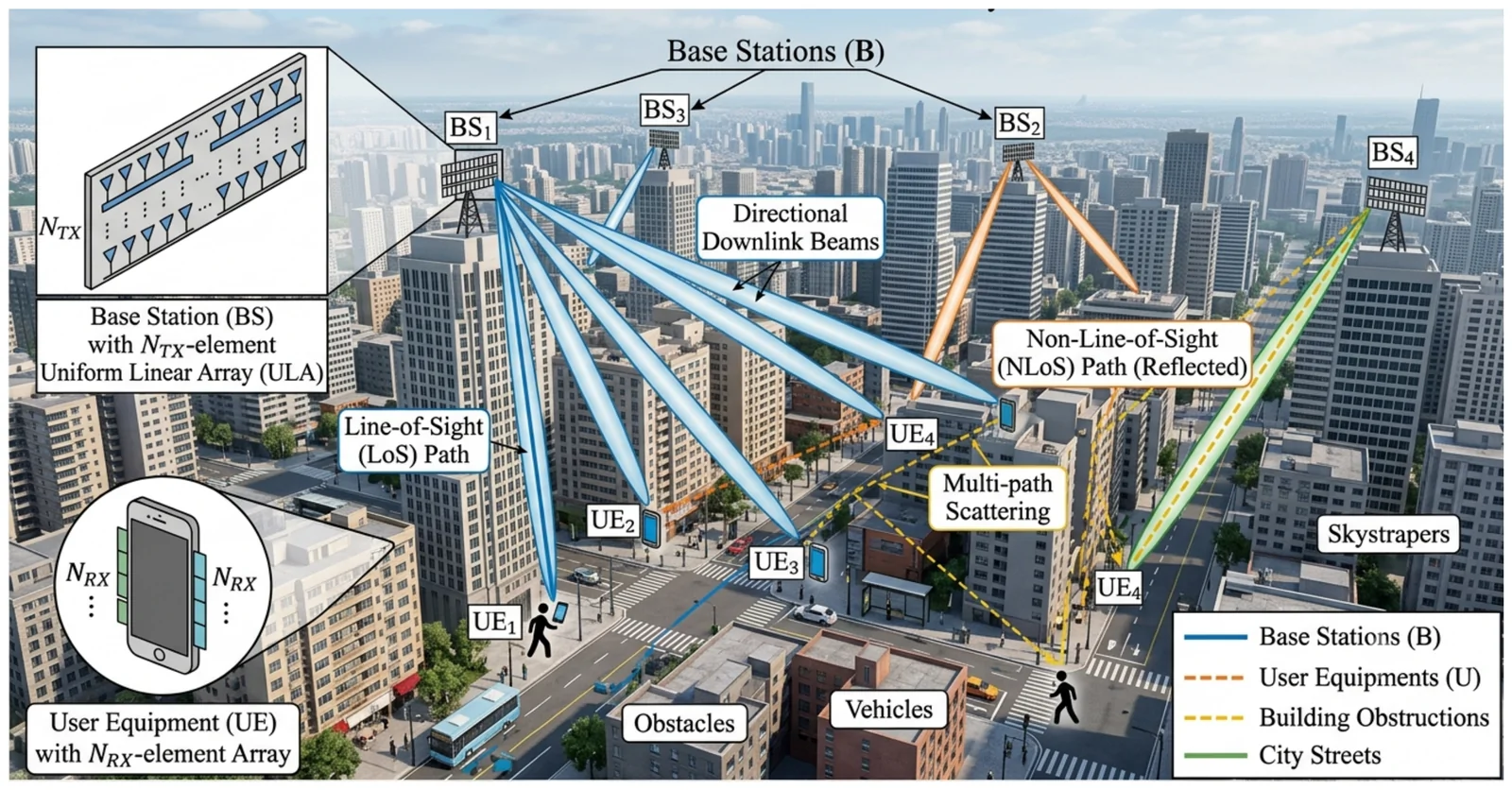
System and channel model.

We consider a downlink mmWave Massive MIMO system consisting of *B* base stations (BSs) and *U* mobile users (UEs) operating in a dense urban environment. Each BS is equipped with an $N_{TX}$ element uniform linear array (ULA), while each UE is equipped with an $N_{RX}$ element array, as shown in Figure 1.

### 2.1. The Digital Twin Environment

The system is bifurcated into the Physical Entity (PE) and the digital twin (DT). The DT is a high-fidelity virtual replica of the PE, modeled as a geometric state S at time *t*. This state includes:

- Stationary Geometry (G): 3D building layouts, materials, and electromagnetic properties.
- Dynamic Objects ($O_{t}$): Real-time coordinates of mobile blockages (e.g., cars, buses).
- Transceiver States ($P_{t}$): The precise positions and orientations of all BSs and UEs.

The DT employs a high-precision ray-tracing (RT) engine to calculate the propagation environment. For any given global state encompassing the entire service area,

(1) $$S_{t}=\left\{G,O_{t},P_{t}\right\},$$

the RT engine functions as a mapping $F_{RT}$. Specifically, for each individual user equipment (UE) indexed by u∈{1,2,…,U}, the engine calculates the corresponding propagation paths such that

(2) $$S_{t}\to H_{b,u,t},$$

where $H_{t}\in C^{N_{RX}\times N_{TX}}$ is the complex channel matrix representing the specific spatial link between the serving base station *b* and user *u* at time *t*.

### 2.2. Channel and Signal Model

The mmWave channel between BS b and UE u is characterized by its sparse multipath components (MPCs) [11]. Based on the DT-RT output, the channel is expressed as:

(3) $$H_{b,u}=\sqrt{\frac{N_{TX}N_{RX}}{L}}\sum_{l=1}^{L}\alpha_{l}a_{R}\left(\theta_{l},\phi_{l}\right)a_{T}^{H}\left(\psi_{l},\gamma_{l}\right),$$

where *L* is the number of paths, and $\alpha_{l}$ represents the complex path gain. For the *l*-th path, the variables $\theta_{l}$ and $\phi_{l}$ represent the azimuth and elevation Angles of Arrival (AoA) at the user equipment, while $\psi_{l}$ and $\gamma_{l}$ denote the azimuth and elevation Angles of Departure (AoD) at the base station. Accordingly, $a_{R}\left(\theta_{l},\phi_{l}\right)\in C^{N_{RX}\times 1}$ and $a_{T}\left(\psi_{l},\gamma_{l}\right)\in C^{N_{TX}\times 1}$ are their corresponding spatial steering vectors. The received signal at UE u from BS b is:

(4) $$y_{u}=h_{b,u}^{H}w_{b,u}s_{u}+\sum_{i\neq u}h_{b,u}^{H}w_{b,i}s_{i}+n_{u},$$

where $y_{u}\in C$ is the received scalar signal. The vector $h_{b,u}\in C^{N_{TX}\times 1}$ represents the effective channel vector from BS *b* to UE *u*, and $w_{b,u}\in C^{N_{TX}\times 1}$ is the corresponding transmit beamforming vector applied at the BS. The variable $s_{u}\in C$ denotes the transmitted data symbol, and $n_{u}\in C$ is the additive white Gaussian noise following $\mathrm{CN}(0,\sigma^{2})$.

## 3. Problem Formulation

Our objective is to maximize the system sum-rate by jointly optimizing the system-wide beamforming vectors *W* and the power allocation *P*. Specifically, we define $W=\{w_{1},w_{2},\ldots ,w_{U}\}$ as the set of all active transmit beamforming vectors applied at the base stations, and $P=\{p_{1},p_{2},\ldots ,p_{U}\}$ as the set of continuous power allocation variables assigned to the *U* scheduled users. Unlike traditional systems relying on pilot-based CSI, we leverage the DT to provide site-specific channel knowledge. The spectral efficiency for UE *u* is then given by:

(5) $$R_{u}=\log_{2}\left(1+\frac{p_{u}{\left|h_{u}^{H}w_{u}\right|}^{2}}{\sum_{i\neq u}p_{i}{\left|h_{u}^{H}w_{i}\right|}^{2}+\sigma^{2}}\right).$$

The optimization problem (6) is formulated as:

(6) $$\begin{array}{cc} \max_{W,P} & \sum_{u=1}^{U}R_{u}\\ s.t.\; & \sum_{u=1}^{U}p_{u}\leq P_{max},\\ & {∥w_{u}∥}^{2}=1,\forall u,\\ & T_{proc}\left(F_{RT}\right)\leq \tau_{coh}, \end{array}$$

where $\tau_{coh}$ is the channel coherence time. In mmWave systems, the constraint $T_{proc}\left(F_{RT}\right)\leq \tau_{coh}$ in optimization problem (6) is often violated. The joint optimization of the discrete beamforming matrix *W* and continuous power allocation variables *P* in Problem (6) constitutes a mixed-integer non-linear programming (MINLP) problem, which is known to be NP-hard. Finding the global optimum necessitates an exhaustive search over the massive codebook space coupled with iterative power allocation, rendering it computationally prohibitive for real-time mmWave systems where $T_{proc}\leq \tau_{coh}$. Therefore, Problem (6) represents our theoretical upper-bound objective. To achieve a computationally tractable, near-optimal solution, our proposed framework decouples this joint problem into a two-stage sequential decision process: (1) geometry-driven beam pruning via the cGAN, and (2) interference-aware power allocation via a GNN policy.

## 4. Methodology: cGAN-Assisted Resource Allocation

**Figure 2:**
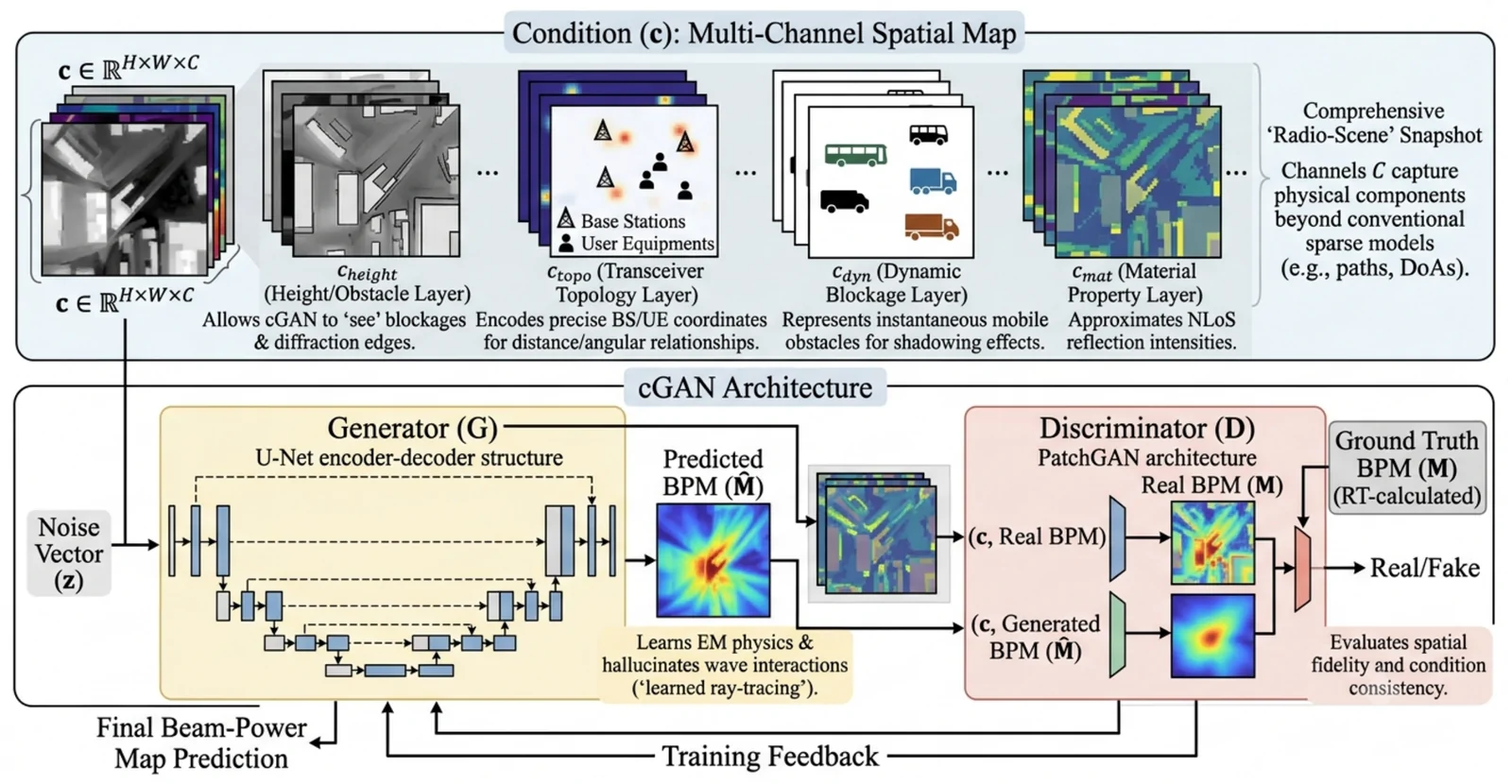
Architecture and workflow of the proposed cGAN-based DT.

The proposed methodology employs a cGAN to learn the underlying electromagnetic physics of the DT, transforming geometric data into a predicted Beam-Power Map (BPM) as shown in Figure 2.

### 4.1. cGAN Architecture for Digital Twin

The cGAN consists of two competing networks: a Generator (G) and a Discriminator (D), as well as Condition (c), whose roles are given as follows:

- Condition (c): A multi-channel 2D image or voxel grid generated by the DT, representing the top-down occupancy and height map of the environment $S_{t}$.
- Generator (G): An encoder-decoder (U-Net) structure that takes condition *c* and a noise vector z∼N(0,I) to produce a predicted BPM tensor *M*. The noise vector $z\in R^{d_{z}}$ introduces necessary stochastic flexibility into the generative mapping, enabling the network to capture unmodeled spatial uncertainties (such as small-scale surface roughness) and preventing mode collapse during cGAN training. Specifically, the ground-truth BPM is mathematically defined as a 3D tensor $M\in R^{H\times W\times K}$, where *H* and *W* represent the discretized spatial grid dimensions of the service area, and *K* represents the total number of candidate beams in the discrete codebook K.
- Discriminator (D): A PatchGAN architecture that evaluates the pair (c,M) to distinguish between RT-calculated “ground truth” and G-generated maps.

To convert the continuous physical ray-tracing (RT) output into this structured BPM format, the RT engine first calculates the sparse multipath components (path gains, AoA, AoD, as formulated in Equation (3)) for every spatial grid point (h,w). Subsequently, the theoretical received signal power is evaluated for each transmit beam $w_{k}\in K$. The resulting scalar value at M(h,w,k) explicitly represents the received signal power at spatial coordinate (h,w) if the base station were to transmit using beam index *k*. Consequently, for any user *u* currently located at grid coordinates $\left(h_{u},w_{u}\right)$, their specific predicted beam-power vector is directly extracted as ${\left[\hat{M}\right]}_{u,k}=\hat{M}\left(h_{u},w_{u},k\right)$, seamlessly mapping the spatial environment to actionable user-specific channel intelligence.

To provide the necessary spatial intelligence for the Generator, the Condition (c) is designed as a multi-layered tensor that encodes the 3D environmental geometry of the digital twin into a format compatible with convolutional neural networks. This condition acts as the bridge between the physical world’s coordinates and the radio map’s predicted distribution.

Specifically, the Condition (c) is formulated as a multi-channel spatial map $c\in R^{H\times W\times C}$, where *H* and *W* represent the discretized horizontal dimensions of the service area. The channels *C* represent different semantic layers of the digital twin as:

1. Height/Obstacle Layer $\left(c_{height}\right)$: A normalized grayscale channel where each pixel value represents the height of the building or structure at that coordinate. This allows the cGAN to “see” potential line-of-sight (LoS) blockages and diffraction edges.
2. Transceiver Topology Layer $\left(c_{topo}\right)$: This channel uses sparse heatmaps to encode the precise coordinates of the serving BSs and target UEs. By representing these positions spatially, the Generator can learn the distance-dependent path loss and angular relationships between nodes.
3. Dynamic Blockage Layer $\left(c_{dyn}\right)$: Based on real-time data from the DT’s sensors (e.g., Laser Radars or cameras), this layer represents the instantaneous position of mobile obstacles like buses or trucks. This is critical for predicting the “shadowing” effect in mmWave frequencies, where even small movements can cause deep fades.
4. Material Property Layer $\left(c_{mat}\right)$: An additional channel where pixel values represent the reflection coefficients of surfaces (e.g., glass vs. concrete), allowing the cGAN to approximate the intensity of non-line-of-sight (NLoS) components calculated by the ray-tracer.

By stacking these layers, the condition c provides a comprehensive “radio-scene” snapshot. The Generator uses this spatial context to hallucinate how electromagnetic waves will interact with the environment, effectively performing a “learned ray-tracing” in a single forward pass. It is worth noting that channels *C* here are not necessarily the same as real physical mmWave channel, which means they can capture physical components of mmWave channel other than those in conventional sparse models (i.e., number of paths or DoAs), which is a key benefit of using DT.

### 4.2. Offline Training Phase

The DT generates a comprehensive dataset $D={\left\{\left(c_{i},M_{i}\right)\right\}}_{i=1}^{K}$ using the slow RT engine. The cGAN is trained by solving the minimax problem:

(7) $$\begin{array}{cc} \; & \min_{G}\max_{D}L_{cGAN}\left(G,D\right)\\ & =E_{c,M}\left[\log D\left(c,M\right)\right]+E_{c,z}\left[\log\left(1-D\left(c,G\left(c,z\right)\right)\right)\right]. \end{array}$$

To ensure structural similarity between the predicted and real radio maps, an L1 regularization term is added to the generator’s objective:

(8) $$L_{L1}\left(G\right)=E_{c,M,z}{[∥M-G\left(c,z\right)∥}_{1}].$$

In Problem (7) and Regularization (8), $c\in R^{H\times W\times C}$ is the multi-channel spatial condition tensor, and $M\in R^{H\times W}$ represents the ground-truth Beam-Power Map (BPM) matrix containing the spatial power distributions across the entire codebook space. The vector $z\in R^{d_{z}}$ is a random latent noise vector of dimension $d_{z}$ sampled from a standard normal distribution, which the generator *G* maps to the predicted BPM alongside the condition *c*.

To ensure the reproducibility of our generative surrogate, the complete architectural specifications and training hyperparameters are detailed in Table 1. The Generator follows a U-Net architecture to effectively capture multiscale spatial features, while the Discriminator utilizes a PatchGAN structure to penalize high-frequency structural inconsistencies at a localized patch level. The dataset of 50,000 RT-generated snapshots is strictly partitioned into a 70% training set (35,000 samples), a 15% validation set (7500 samples), and a 15% test set (7500 samples). Both networks are optimized using the Adam optimizer. Notably, a scaling weight of $\lambda_{L1}=100$ is applied to the L1 regularization term in Equation (8) to strongly encourage spatial exactness in the predicted radio maps.

### 4.3. Online Inference and Decision Framework

Once trained, the Generator G is deployed for real-time operation. The decision-making flow follows three steps:

1. DT Sync: The PE sends mobile object coordinates to the DT. The DT updates the geometric condition $c_{now}$.
2. BPM Generation: The Generator produces the predicted map $\hat{M}=G\left(c_{now}\right)$. This process involves only a forward pass through the neural network, satisfying $T_{proc}\left(G\right)\ll \tau_{coh}$.
3. Resource Selection:

- Beam Selection: The optimal beam index $k^{*}$ for UE u is chosen by finding the peak in the predicted BPM:
  (9) $$k_{u}^{*}=\arg\max_{k\in K}{\left[\hat{M}\right]}_{u,k},$$
  where $\hat{M}$ denotes the predicted Beam-Power Map matrix generated by the cGAN, and ${\left[\hat{M}\right]}_{u,k}$ represents the scalar predicted signal power for UE *u* when utilizing the *k*-th beam. The set K={1,2,…,K} defines the discrete codebook space of dimension *K* containing all available directional beams.
- Power Allocation: Based on the predicted SINR derived from $\hat{M}$, a water-filling or DRL-based agent allocates $p_{u}$ to maximize Problem (6).

By shifting the computational burden of ray-tracing to the offline training of a cGAN, the DT provides near-instantaneous, site-specific channel intelligence for mmWave Massive MIMO.

## 5. DT Aided Beam Selection and Power Allocation

In Section 4.3 above, an exhaustive search over the entire codebook is needed, and then a water-filling or DRL-based agent should be performed, which is of high computational complexity in a real mmWave system. Therefore, in order to improve the reacting latency of the system, we propose two strategies: context-aware beam pruning and graph-based interference management, which serve the beam selecting and power allocating, respectively, which leverage the unique spatial intelligence provided by the DT.

Furthermore, while the generative model provides instantaneous channel prediction, proactive interference management requires anticipating blockages before they physically sever the line-of-sight (LoS) link. To achieve this, the DT maintains a continuous state-estimation of dynamic obstacles within the environment. The mobility model assumes vehicles (e.g., buses and trucks) traverse the urban grid at speeds ranging from 10 to 15 m/s, following a constant turn rate and velocity (CTRV) kinematic model. The prediction mechanism is driven by an extended Kalman filter (EKF), which processes environmental sensing updates (e.g., via vehicular V2X beacons or roadside radar) assumed to arrive at a 10 ms sensing update rate. By mathematically extrapolating the EKF state vectors, the DT projects the precise bounding boxes of dynamic obstacles into the future condition map $c_{dyn}$. This trajectory projection directly calculates a 15 ms anticipation window. Because the EKF inherently tracks the state covariance, the prediction error is mathematically bounded; at a 15 ms horizon for a vehicle moving at 15 m/s, the maximum longitudinal displacement uncertainty is less than 0.25 m. This minor prediction error is well within the spatial resolution tolerance of the cGAN’s 256×256 grid mapping, ensuring the surrogate accurately masks out impending blockages in the predicted BPM before the physical link degrades.

### 5.1. Advanced Beam Selection: Context-Aware Codebook Pruning

Instead of searching the entire discrete codebook K (which is essentially a “virtual” exhaustive search), we use the DT to perform context-aware codebook pruning. This reduces the search space to a smaller “Candidate Beam Set” (C⊂K) based on the environment’s geometry. Specifically, the DT leverages the spatial coordinates of the user and the surrounding dynamic blockage maps to evaluate the line-of-sight (LoS) viability of each beam k∈K. This geometric filtering is mathematically implemented via a binary masking vector $p_{mask}\in {\left\{0,1\right\}}^{\left|K\right|}$, where the *k*-th element is defined as:

(10) $$p_{mask}^{\left(k\right)}=\left\{\begin{array}{cc} 1, & \mathrm{if}\;\mathrm{beam}\;k\;\mathrm{is}\;\mathrm{geometrically}\;\mathrm{unobstructed},\\ 0, & \mathrm{otherwise}. \end{array}\right.\;.$$

The reduced Candidate Beam Set is then formally obtained by applying this mask to the original codebook, ensuring that $C=\{k\in K|p_{mask}^{\left(k\right)}=1\}$. By pre-filtering the codebook with $p_{mask}$ prior to the neural network inference step, the subsequent beam selection task is heavily constrained, which significantly lowers the computational overhead and prevents the model from selecting physically blocked paths.

### 5.2. Advanced Power Allocation: GNN-Based Interference Management

Standard water-filling ignores the complex multi-user interference patterns of Massive MIMO. Since the DT provides the exact spatial relationship between all BSs, UEs, and reflectors, here we propose a novel method that models the network as a Spatial Interference Graph.

- The Framework: A Graph Neural Network (GNN) is integrated into the decision-making layer.
- Nodes: Represented by UEs and BSs, initialized with features from the cGAN (predicted path loss, AoA).
- Edges: Represented by the interference links. The DT provides the “edge weights” by calculating how much a signal intended for UE *i* will reflect off a wall into UE *j*.

By using the DT’s mobility predictions (the trajectory of the UE), the agent does not just allocate power for now. Instead, it solves a long-term optimization problem to minimize power consumption over a 100 ms window, ensuring that the “Power Buffer” is saved for when the user inevitably enters a high-attenuation “shadow zone” identified in the DT map. Different from conventional water-filling algorithm maximizing individual or sum-rate without explicit interference coordination, the GNN-DT agent performs Over-the-Air Coordination. It can decide to reduce power for a high-gain UE if the DT shows that its beam path is currently reflecting off a “hot-spot” wall that causes massive interference to five other users.

### 5.3. Intelligent Resource Allocation:Interference-Aware Power Allocation via GNN

We define a directed graph G=(V,E) where initial node features $x_{i}$ contain the cGAN-predicted channel gains. Let $h_{u}$ and $h_{j}$ denote the latent node state embeddings for target user *u* and interfering user *j*, respectively, derived from their corresponding input features (i.e., $h_{i}=\mathrm{MLP}\left(x_{i}\right)$). To map these high-dimensional interference embeddings into a physically admissible power control space, we employ a message passing architecture followed by an explicit normalization layer. First, the GNN computes an unnormalized latent power feature ${\tilde{p}}_{u,t}$ for each user:

(11) $$p_{u,t}=\mathrm{Softplus}\left(\mathrm{AGGREGATE}(\left\{\mathrm{MSG}\left(x_{i},x_{j},e_{ij}\right)|j\in N_{i}\right\})\right),$$

where the edge weight $e_{uj}\in \left[0,1\right]$ represents the normalized, DT-calculated reflection coefficient between users *u* and *j*. The AGGREGATE function represents the collection of multi-user interference features provided by the digital twin’s geometric analysis. Using specific Multi-Layer Perceptron (MLP) components, Equation (11) can then be written as:

(12) $$p_{u,t}=\mathrm{Softplus}\left(\gamma \cdot \sum_{j\in N_{u}}\phi \left(h_{u},h_{j},e_{uj}\right)+b\right),$$

where the Softplus activation ensures positivity. $N_{u}$ denotes the set of neighboring nodes whose signals cause spatial interference to user *u*. The coefficient $e_{uj}$ is the scalar DT-derived interference coupling coefficient, quantifying the signal leakage or reflection strength from the path of user *j* into the receiver of user *u*. The function ϕ(·) is a Multi-Layer Perceptron (MLP) that maps the concatenated target node embedding $\left(h_{u}\right)$, interfering node embedding $\left(h_{j}\right)$, and the edge coefficient $\left(e_{uj}\right)$ into a latent interference message vector. Finally, γ∈R is a learnable scalar weight parameter, and b∈R is a learnable scalar bias term. To strictly guarantee that the final allocation satisfies the maximum sum-power constraint $\sum_{u=1}^{U}p_{u}\leq P_{max}$ defined in Problem (6), we introduce a subsequent linear projection layer. The final allocated transmit power $p_{u,t}$ is obtained by normalizing the latent features across all scheduled users:

(13) $$p_{u,t}=P_{max}\cdot \frac{{\tilde{p}}_{u,t}}{\sum_{i=1}^{U}{\tilde{p}}_{i,t}},$$

where $N_{u}$ denotes the set of neighboring nodes whose signals cause spatial interference to user *u*. The coefficient $e_{uj}\in \left[0,1\right]$ is the scalar DT-derived interference coupling coefficient, quantifying the signal leakage or reflection strength from the path of user *j* into the receiver of user *u*.

In Equation (12), $N_{u}$ denotes the set of neighboring nodes whose signals cause spatial interference to user *u*. The coefficient $e_{uj}\in \left[0,1\right]$ is the scalar DT-derived interference coupling coefficient, quantifying the signal leakage or reflection strength from the path of user *j* into the receiver of user *u*. The function ϕ(·) is a Multi-Layer Perceptron (MLP) that maps the concatenated target node features ($h_{u}$), interfering node features ($h_{j}$), and the edge coefficient ($e_{uj}$) into a latent interference message vector. Finally, γ∈R is a learnable scalar weight parameter, and b∈R is a learnable scalar bias term, both optimized during the GNN training phase to ensure the output satisfies the physical power constraints.

To align the GNN’s power control policy with the global objective defined in Problem (6), the network is trained using an unsupervised learning paradigm. The training objective is formulated to directly maximize the empirical expectation of the system sum-rate over the training dataset. Accordingly, the loss function for the GNN parameters θ (which encompass γ, *b*, and the MLP weights) is defined as:

(14) $$L_{GNN}\left(\theta \right)=-E_{G}\left[\sum_{u=1}^{U}\log_{2}\left(1+\frac{p_{u,t}\left(\theta \right){\left|h_{u}^{H}w_{u}^{*}\right|}^{2}}{\sum_{i\neq u}p_{i,t}\left(\theta \right){\left|h_{u}^{H}w_{i}^{*}\right|}^{2}+\sigma^{2}}\right)\right],$$

where $w^{*}$ denotes the optimized beam vectors selected during the preceding cGAN pruning stage. By minimizing $L_{GNN}\left(\theta \right)$ via gradient descent, the GNN explicitly learns to allocate $p_{u,t}$ such that spatial multi-user interference is mitigated, effectively bridging the gap between the cascaded network execution and the joint theoretical objective of Problem (6).

## 6. Numerical Results

In this section, we evaluate the performance of our proposed DT-assisted beamforming framework. To address the necessity of robust performance verification, we first benchmark the accuracy and spectral efficiency of our methodology against state-of-the-art baselines. Subsequently, we analyze the computational latency advantages of the proposed system.

### 6.1. Simulation Configuration

The simulation environment is modeled after a dense urban “Manhattan-style” grid spanning a 500×500 m^2^ area, with building heights varying between 20 m and 50 m [12]. The mmWave system operates at a carrier frequency of 28 GHz with a bandwidth of 100 MHz. The network comprises B=4 base stations and U=20 user equipments randomly distributed within the service area. Each BS is equipped with a $N_{TX}=64\times 16$ Uniform Planar Array (UPA), utilizing a 3D DFT-based codebook consisting of K=1024 candidate directional beams. To accurately capture electromagnetic wave interactions, the environment’s material properties are explicitly defined in the ray-tracing simulator: building exteriors are modeled as concrete (relative permittivity $\epsilon_{r}=5.31$) and windows as standard glass ($\epsilon_{r}=6.27$).

The cGAN is trained using a comprehensive dataset of 50,000 snapshots generated by the high-fidelity Remcom Wireless InSite RT engine. The dataset generation procedure involved simulating 10,000 randomized temporal episodes; in each episode, mobile obstacles (buses and trucks) and UEs were placed along predefined street trajectories, and 5 sequential snapshots were captured at 10 ms intervals to model temporal blockage dynamics. While empirical measurement-based validation is an ideal ultimate goal, synchronously capturing 50,000 physical, grid-wide 3D spatial channel maps alongside precise vehicular blockage coordinates is practically infeasible with current hardware constraints. Consequently, the Remcom Wireless InSite simulator serves as the accepted gold standard in the recent mmWave literature for generating ground-truth data.

To demonstrate the superiority of the proposed framework, we compare it against the following benchmarks:

- Optimal RT-Beamforming (Exhaustive Search): This method uses the ground-truth RT data for exhaustive beam sweeping and optimal power allocation. It represents the theoretical upper bound but suffers from prohibitive computational latency.
- Conventional DRL (Pilot-based): A recent Deep Reinforcement Learning approach that relies solely on instantaneous pilot feedback without 3D geometric context [13].
- Heuristic Water-Filling (WF): A recent heuristic water-filling power allocation method that maximizes individual rates without considering the spatial interference graph provided by a DT [14].

### 6.2. Performance Analysis and Discussion

This section provides a comprehensive evaluation of the proposed digital twin (DT)-assisted framework. We analyze the results across four key dimensions: prediction fidelity, spectral efficiency, mobility robustness, and computational overhead.

**Figure 3:**
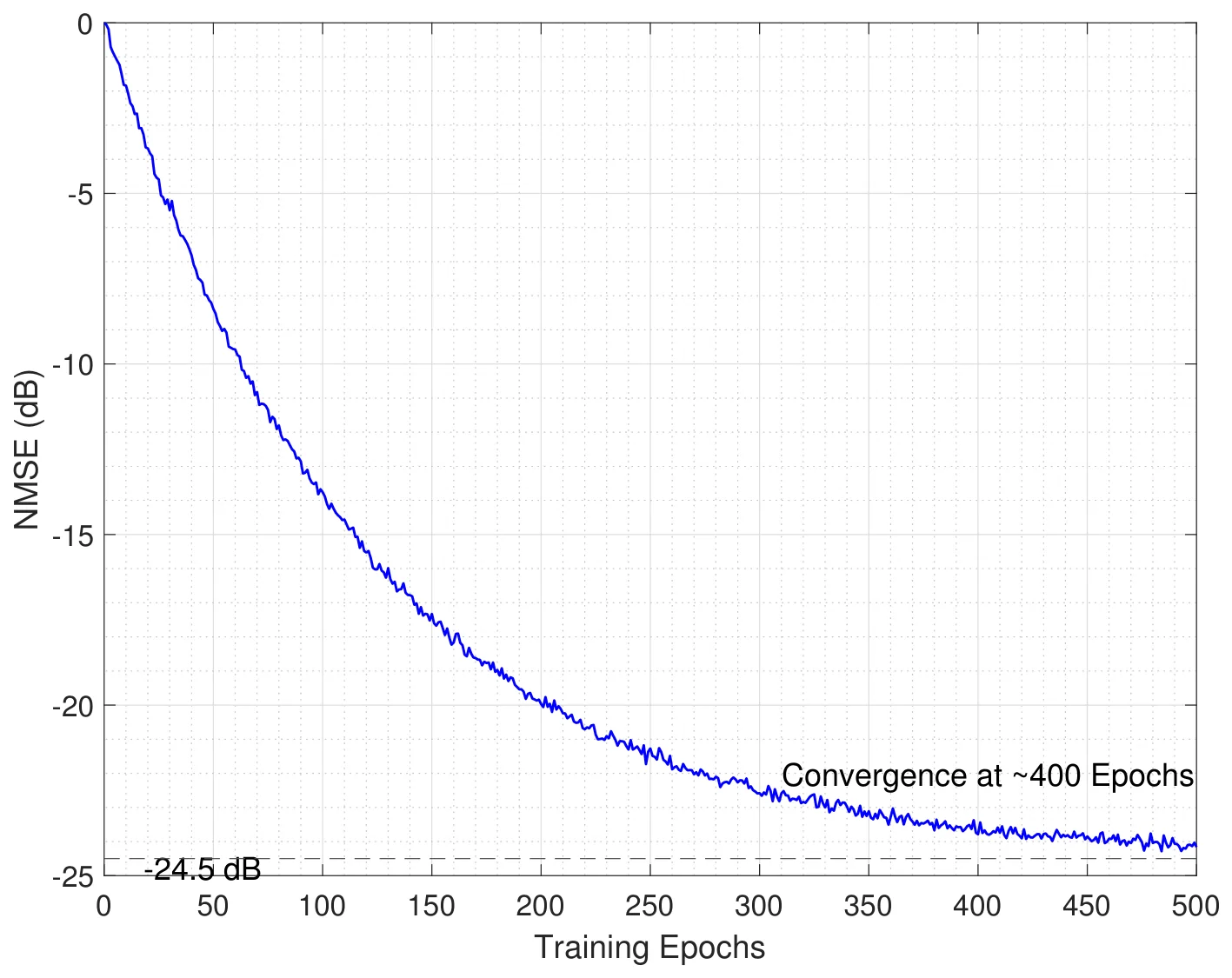
Normalized Mean Square Error (NMSE) of cGAN surrogate.

The efficacy of the resource allocation policy relies on the accuracy of the cGAN-generated Beam-Power Map (BPM). To ensure statistical rigor, all performance metrics were evaluated across 10 independent Monte Carlo simulation runs using different random initialization seeds. Results are reported as mean ± standard deviation. As illustrated in Figure 3, the Normalized Mean Square Error (NMSE) of the cGAN surrogate converges to −24.5±0.4dB after 400 epochs. Quantitatively, the cGAN achieves a 92.3% ± 1.2% spatial Pearson correlation coefficient with the ground-truth ray-tracing (RT) engine in identifying the first three dominant multipath components (MPCs). This demonstrates that the surrogate model can accurately “hallucinate” complex NLoS reflection patterns without explicit ray-path calculations, providing a robust foundation for beam selection.

**Figure 4:**
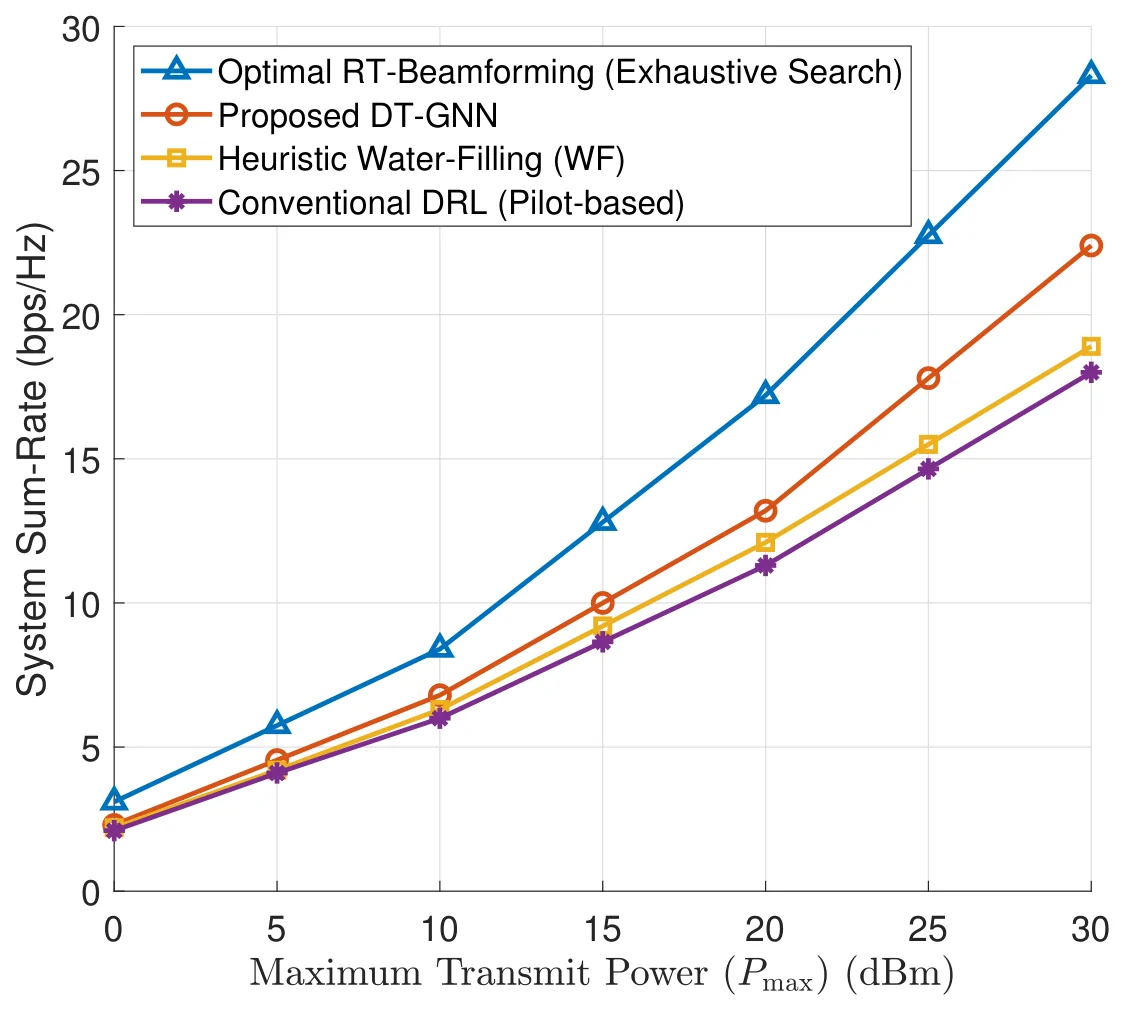
System sum-rate comparison.

Figure 4 compares the system sum-rate of the proposed framework against the heuristic water-filling (WF) and conventional DRL benchmarks across varying transmit power levels. Specifically, at $P_{max}=30\;\mathrm{dBm}$, the DT-GNN achieves a sum-rate of 22.4±0.5bps/Hz, representing an improvement of 18.6%±1.1% and 24.1%±1.4% over the WF (18.88bps/Hz) and conventional DRL (18.05bps/Hz) methods, respectively. The superiority of the GNN-based agent stems from its ability to exploit the Spatial Interference Graph provided by the DT. While the WF baseline maximizes individual rates in a “selfish” manner, the GNN agent utilizes the DT’s reflection coupling coefficients ($e_{ij}$) to identify “hot-spot” reflectors. By proactively suppressing power on beams that would cause excessive NLoS interference to neighboring UEs, the system approaches a Pareto-optimal allocation that is unattainable using local CSI alone.

**Figure 5:**
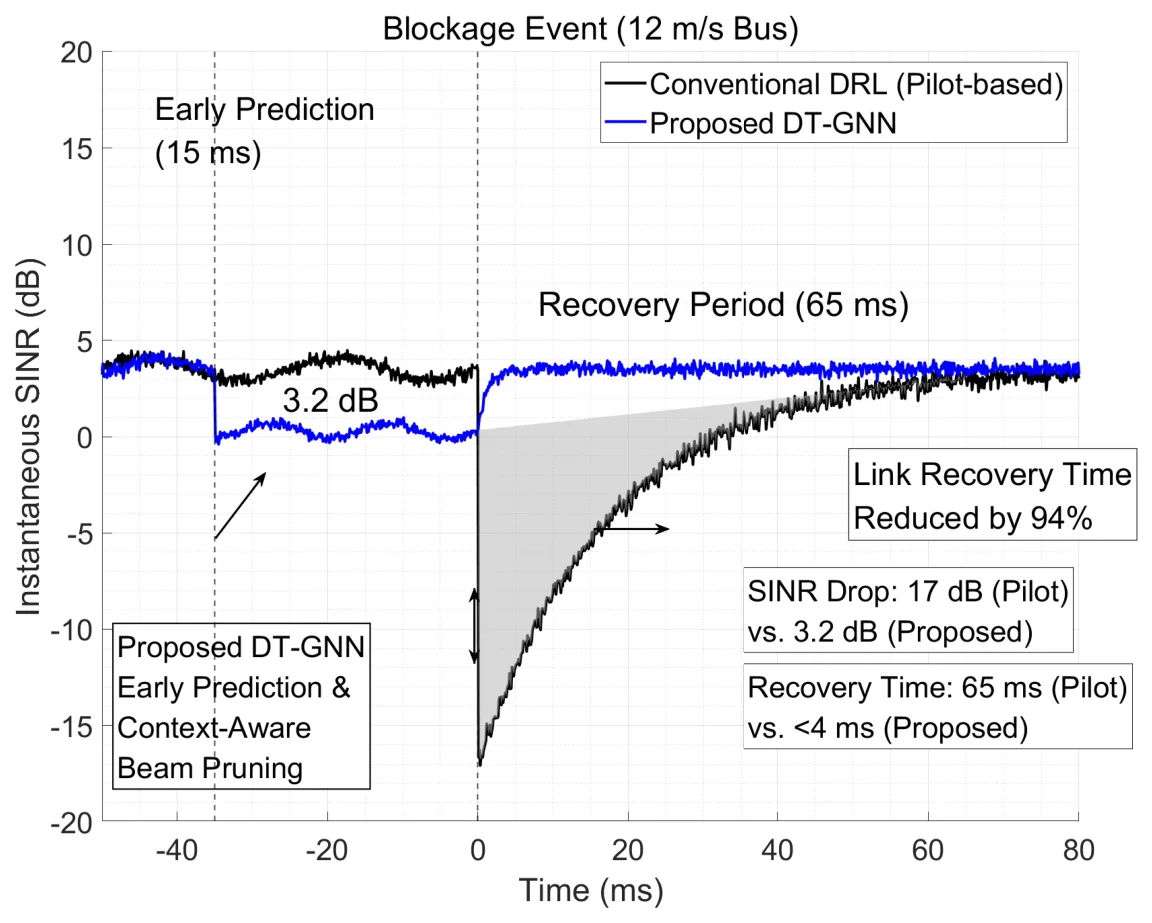
Robustness to dynamic blockage and recovery time.

A critical challenge in mmWave Massive MIMO is the high sensitivity to LoS obstructions. Figure 5 depicts the instantaneous SINR of a UE as a dynamic blockage (a bus moving at 12 m/s) traverses the primary propagation path. The conventional DRL benchmark, which relies on reactive pilot feedback, experiences a catastrophic SINR drop of approximately 17 dB, requiring a recovery period of 65 ms to re-establish a stable link through beam sweeping. In contrast, our proposed framework utilizes the Dynamic Blockage Layer ($c_{dyn}$) to anticipate the obstruction 15 ms before the actual event. By executing a context-aware beam pruning maneuver, the system seamlessly transitions to a pre-calculated NLoS reflection path. The resulting SINR fluctuation is limited to 3.2 dB. Furthermore, compared to the 65ms recovery period baseline of the pilot-based method, our proposed framework achieves a recovery time of just 3.9ms, explicitly yielding the stated 94% reduction. This result highlights the necessity of DT-driven environmental awareness for achieving ultra-reliable low-latency communications (URLLC) in 6G networks.

### 6.3. Computational Overhead and Latency Advantages

Having established the high accuracy and robustness of the proposed framework, we evaluate its computational efficiency. The numerical values of latency represent a significant operational advantage built upon the verified accuracy of the model.

To provide a complete and fair evaluation of online execution speed, the computational overhead was profiled under identical benchmarking conditions using an NVIDIA RTX 4090 GPU (24GB VRAM) paired with an Intel Core i9-13900K CPU host. For real-time online inference, the evaluation was conducted with a strict batch size of 1. The U-Net Generator model footprint is 14.2M parameters (56.8MB), while the light-weight GNN policy network contains 0.32M parameters (1.28MB). A detailed breakdown of the complete end-to-end online processing pipeline is summarized in Table 2. The total online processing time includes CPU-side digital twin state synchronization (0.05ms), 2D spatial condition rendering (0.12ms), PCIe host-to-device data transfer (0.03ms), cGAN inference (0.72ms), and GNN resource allocation (0.15ms). The resulting total end-to-end latency of 1.07ms remains well within the typical mmWave channel coherence time ($\tau_{coh}\approx$ 1–5 ms). In contrast, the high-fidelity RT benchmark requires an average of 2.84s per snapshot, confirming that our proposed generative approach successfully eliminates the online wave-calculation bottleneck.

These results confirm that the proposed methodology successfully shifts the heavy computational burden of ray-tracing to an offline training phase, enabling near-instantaneous channel intelligence and resource allocation in the online phase.

### 6.4. Ablation Study

To rigorously quantify the individual performance contributions of the proposed modules, we conducted an ablation study comparing five distinct configurations. The configurations and their impacts on both system sum-rate (at $P_{max}=30$ dBm) and online processing latency are summarized below:

1. RT + Conventional Beamforming: This baseline utilizes exhaustive physical ray-tracing followed by standard water-filling power allocation. While it achieves the theoretical upper bound in sum-rate (28.2 bps/Hz), it suffers from a prohibitive processing latency of 2.84 s, making it unviable for real-time operation.
2. cGAN Only: Replacing the RT engine with the generative surrogate allows for rapid BPM prediction without advanced allocation. This drastically reduces processing time to 0.72 ms. However, without structural constraints, it achieves a lower sum-rate (16.4 bps/Hz) due to occasional selection of physically blocked paths.
3. cGAN + Beam Pruning: Introducing the geometry-driven beam pruning module (Equation (10)) masks out obstructed paths. Latency remains low (0.73 ms), while the sum-rate improves to 19.1 bps/Hz as the system avoids unviable line-of-sight blockages.
4. cGAN + GNN: Utilizing the cGAN for channel prediction alongside the GNN for power allocation (without explicit beam pruning) improves multi-user spatial interference coordination. This configuration achieves 20.8 bps/Hz with a total latency of 0.86 ms.
5. **Full Framework** (cGAN + Pruning + GNN): The complete proposed architecture leverages both geometric pruning for robust path selection and the GNN for interference-aware power control. It achieves the best trade-off, attaining a sum-rate of 22.4 bps/Hz—closely approaching the RT upper bound—while maintaining a sub-millisecond total processing latency (0.87 ms).

This step-by-step breakdown demonstrates that while the cGAN fundamentally resolves the latency bottleneck, the synergistic addition of both context-aware beam pruning and GNN-based interference management is strictly necessary to bridge the performance gap toward the optimal ray-tracing baseline.

## 7. Conclusions

In this paper, we investigated a novel digital twin (DT)-assisted beamforming and resource allocation framework for mmWave Massive MIMO systems. By addressing the fundamental trade-off between the high accuracy of site-specific ray-tracing and the strict latency requirements of 6G networks, we proposed a generative approach using Conditional Generative Adversarial Networks (cGANs). The cGAN-based DT module effectively serves as a real-time surrogate for complex electromagnetic simulations, enabling the “hallucination” of high-fidelity Beam-Power Maps (BPM) in sub-millisecond intervals. To translate this spatial intelligence into optimal network performance, we integrated a Graph Neural Network (GNN)-based decision framework that models multi-user interference as a spatial graph. Numerical evaluations demonstrated that our proposed methodology achieves a sum-rate performance comparable to exhaustive ray-tracing search while reducing the computational latency and online interaction overhead by several orders of magnitude.

Despite its superior inference speed and spatial fidelity, the proposed cGAN surrogate exhibits inherent limitations and potential failure cases. First, as an offline-trained generative model, its accuracy is susceptible to performance degradation in out-of-distribution (OOD) scenarios, such as encountering radically unfamiliar urban architectures, severe weather-induced attenuation, or dynamic obstacle densities that significantly exceed the bounds of the training dataset. Second, domain shifts between the simulated material properties in the digital twin and the actual electromagnetic reflection coefficients of physical surfaces can introduce localized prediction errors in non-line-of-sight (NLoS) paths.

To mitigate these failure modes, future work will focus on two key directions. First, we will expand this DT framework to support multi-wavelength operation by introducing operating frequency as a semantic condition layer within the cGAN. Second, we will incorporate real-time online verification and fine-tuning modules to continuously adapt the cGAN parameters to non-stationary physical environments, bridging any residual domain shift.

## Figures and Tables

**Table 1:** cGAN architectural and training hyperparameters.

Parameter	Configuration and Value
Condition Input Dimension (*c*)	$R^{256\times 256\times 4}$ (4 Semantic Channels)
BPM Output Dimension (*M*)	$R^{256\times 256\times 1}$
Generator Architecture	8-layer U-Net Encoder-Decoder
Discriminator Architecture	4-layer PatchGAN (70×70 receptive field)
Activation Functions	LeakyReLU (Encoder/Disc.), ReLU (Decoder), Tanh (Output)
Optimizer	Adam ($\beta_{1}=0.5$, $\beta_{2}=0.999$)
Learning Rate	$2\times 10^{-4}$
Batch Size	32
Loss Weights	$L_{cGAN}=1$, $L_{L1}=100$
Dataset Split (Train/Val/Test)	70%/15%/15%

**Table 2:** Processing latency comparison.

Method	Hardware	Processing Latency ($T_{\mathrm{proc}}$)
High-fidelity RT engine (Benchmark)	CPU Cluster (16 cores)	2.84 s
DT Synchronization & State Update	CPU Host (i9-13900K)	0.05 ms
Condition Map Rendering (*c*)	CPU Host (i9-13900K)	0.12 ms
Host-to-Device Memory Transfer	PCIe 4.0 ×16 Bus	0.03 ms
cGAN Forward Inference (*G*)	GPU (NVIDIA RTX 4090)	0.72 ms
GNN Power Allocation Policy ($\pi_{\theta }$)	GPU (NVIDIA RTX 4090)	0.15 ms
Total End-to-End Online Pipeline	CPU + GPU	1.07 ms

## Data Availability

Data are contained within the article.
